# Novel rapid method for the characterisation of polymeric sugars from macroalgae

**DOI:** 10.1007/s10811-016-0995-0

**Published:** 2016-11-16

**Authors:** S. E. Spicer, J. M. M. Adams, D. S. Thomas, J. A. Gallagher, Ana L. Winters

**Affiliations:** 1 MicroPharm Ltd., Newcastle Emlyn, Carmarthenshire SA38 9BY UK; 2 Institute of Biological, Environmental and Rural Sciences (IBERS), Aberystwyth University, Gogerddan, Aberystwyth, SY23 3EE UK

**Keywords:** Seaweed, Laminarin, HPAEC-Pad, Nutraceuticals, Biorefining

## Abstract

Laminarins are storage polysaccharides found only in brown seaweeds, specifically Laminarialaes and Fucales. Laminarin has been shown to have anti-apoptotic and anti-tumoural activities and is considered as a nutraceutical component that can positively influence human health. The structure is species dependent, generally composed of linear ß(1–3) glucans with intrachain β(1–6) branching and varies according to harvest season and environmental factors. Current methods for analysis of molar mass and DP length are technically demanding and are not widely available. Here, we present a simple inexpensive method which enables rapid analysis of laminarins from macroalgal biomass using high-performance anion exchange chromatography with pulsed amperometric detection (HPAEC-PAD) without the need for hydrolysis or further processing. This is based on the linear relationship observed between log_10_ DP and retention time following separation of laminarins on a CarboPac PA-100 column (Dionex) using standard 1,3-β-d-gluco-oligosaccharides ranging in DP from 2 to 8. This method was applied to analyse laminarin oligomers in extracts from different species harvested from within the intertidal zone on Welsh rocky shores containing laminarin polymers with different ranges of DP. The degree of polymerisation and extrapolated molar mass agreed well with values estimated by LC-ESI/MS^*n*^ analysis and those reported in the literature.

## Introduction

Marine algal biomass is both renewable and carbon neutral and therefore an attractive alternative substrate for the production of biofuel and potential biorefinery products (Mazumdar et al. 2014) including biologically active polysaccharides (Menshova et al. 2014). Brown seaweeds of the phylum Ochrophyta include species that cover all intertidal zones. Species range from *Laminaria hyperborea* and *L. digitata* in the low intertidal zone where organisms are covered with seawater for all but the lowest of tides to *Pelvetia canaliculata*, which is found high on the shore and can survive for many days out of water (Fernandez-Marin et al. 2011). The most abundant polysaccharides of the brown algal species include laminarins, fucoidans and alginic acids which combined are reported to account for 40–80% of the dry defatted algal biomass (Zvyagintseva et al. 2003). Detailed structures of individual algal compounds have only been identified in a few cases, but it is known that structures are complex with much variation between species. Variations in composition may also depend on the season and climatic conditions when harvested and also the extraction procedures used (Pomin and Mourao 2008; Adams et al. 2011). Laminarins (also known as laminarans or leucosin) (Alderkamp et al. 2007) are a group of polysaccharides found in large quantities in brown algae. These storage carbohydrates are β(1,3) linked β-d-glucans with β(1–6) intrachain branching. Average degree of polymerisation (DP) is reported in the literature as 25 with a molecular mass of up to 5 kDa (Alderkamp et al. 2007; Rioux et al. 2007, 2010; Zvyagintseva et al. 2005). There are two subdivisions of laminarins: M-chain types have a terminal mannitol moiety whereas G-chain types have a terminal glucose residue. There are variations in structures and ratios of M-type to G-type between seaweed species; these can vary according to the season and other environmental factors such as the amount of nutritive salts available and age of the frond. In a study of *Saccharina* (*Laminaria*) *longicruris*, harvested from a temperate region in Eastern Canada, it was found that the crude laminarin content was significantly higher in samples taken in May and August of 2005 when compared with November 2005 and June 2006. The differences were ascribed to the difference in amounts of nutritive material available in the water and/or frond age and stage of growth cycle with a decline in winter months attributed to utilisation of laminarin as a carbon source (Rioux et al. 2009, 2010). Laminarins are found in the vacuoles of algal cells and may be released into the pelagic system following cell lysis or grazing of the plants by marine organisms. They are rapidly degraded in the marine system, one route being as a substrate for marine bacteria (Alderkamp et al. 2007). The level of solubility of the polymers is dictated by the degree of branching; the highly branched oligomers being soluble in cold water with the less-branched isomers only soluble in warm water (Rioux et al. 2007).

The identification of novel bioactive natural compounds has become an important area of oncotherapeutic and immunopharmacological research (Ermakova et al. 2014). Laminarin has been shown to have antiapoptotic and antitumoural activities and is considered as a nutraceutical component that can positively influence human health (Rioux et al. 2010). The β(1,3) linked β-d-glucan structure of laminarins are similar to that of curdlan, an extracellular bacterial glucan with a simple homopolymeric, unbranched (1→3)-β-glucan structure. Curdlan has been chemically modified to create a large variety of derivatives intended for food industry and biomedical applications (Zhang and Edgar 2014). The degree of polymerisation of laminarins has been shown to have an effect on anticancer bioactivity, with a mixture of DP of 9–23 showing a higher effect on cell lines than polysaccharides with a range of 4–13 glucose residues (Menshova et al. 2014). Laminarins as neutraceuticals therefore have the potential to become high-value coproducts of a macroalgal biorefinery. Previously, the DP profiles of laminarin have been determined through matrix-assisted laser desorption/ionisation-time of flight (MALDI-TOF) analysis, with permethylated derivatives of laminarins from different species of brown algae (Chizhov et al. 1998). The method described here has been developed from a technique developed to profile fructose polymers (fructans) (Andrew Cairns, personal communication) as a rapid and simple technique for determining the DP range of (1→3)-β-glucan polymers in macroalgae. This method does not require samples to be derivatised or hydrolysed prior to analysis and thus can be applied to polysaccharide extracts to inform its further use as a nutraceutical product.

## Materials and methods

### Extraction of laminarin from biomass

Macroalgae species from the sublittoral fringe were harvested and stored on ice prior to freezing at −20 °C within 2 h of harvest. Six species of seaweed were chosen to give biomass from a range of intertidal zones. *Laminaria hyperborea* was harvested from the low tide zone, *Fucus serratus* from the lower mid shore, *Ascophyllum nodosum* and *Fucus vesiculosus* from the mid shore, *Fucus spiralis* from the upper mid shore and *Pelvetia canaliculata* from the high tide zone. *Laminaria hyperborea*, *P. canaliculata* and *F. spiralis* were harvested from Tresaith, Ceredigion (OS Grid Ref SN279517) in August 2011. *A. nodosum*, *F. serratus* and *F. vesiculosus* were harvested from Aberystwyth, Ceredigion (OS Grid Ref SN583823) in August 2010. Samples were freeze dried and milled and water-soluble polysaccharides were isolated as described by Rioux et al. (2007). Dried and finely milled seaweed (30 g) was initially treated to remove pigments with the addition of 200 mL of 85% ethanol. The mixture was incubated at room temperature for 5 h with constant stirring, centrifuged (8500×*g*) and the pellet retained. This step was repeated twice with the pellet material at an incubation temperature of 70 °C and resulted in a pellet of depigmented seaweed material which included precipitated polymeric sugars and denatured proteins. To precipitate dissolved alginates and anionic fucoidans from the solution, the pellet was stirred in 200 mL of 2% (*w*/*v*) calcium chloride solution at 70 °C for 4 h and then centrifuged (8500×*g*). The supernatant was retained and pooled with supernatant from a second calcium chloride extraction, as before, of the pellet. Combined supernatants contained mostly neutral laminarins. Laminarin suspensions were filtered through 1.2 μm GF/C filters (Whatman) under reduced pressure to removed suspended solid macroalgal material, and then reduced in volume by evaporation under reduced pressure at 40 °C to give a volume of 20 mL. Polysaccharides were precipitated from the calcium chloride solution by the addition of 180 mL of ethanol. The mixture was cooled for 2 h at −20 °C then centrifuged at 8500×g in preweighed containers. The pellet was retained and dried under a stream of nitrogen and freeze dried overnight.

### Analysis of laminarin extracts using high-performance anion exchange chromatography with pulsed amperometric detection (HPAEC-PAD)

Crude laminarin fractions were analysed using Dionex high-performance anion exchange chromatography with pulsed amperometric detection (HPAEC-PAD). HPAEC-PAD analysis was performed on a Dionex system consisting of LC30 chromatography oven, GP40 pump and AS autosampler (Dionex, USA). An ED50 electrochemical detector in integrated amperometry mode with AgCl reference electrode, and conventional permanent gold electrode was used with Chromeleon 6.80 software (Dionex) to detect carbohydrates. The waveform used was the standard carbohydrate quadruple waveform recommended for use with CarboPac columns. Separation of compounds was carried out using a CarboPac PA-100 column 250 mm × 4 mm column fitted with PA-100 guard column (50 mm × 4 mm) (Dionex). Solutions of laminarin extracts in water were prepared at concentrations of 10 mg mL^−1^. Solvents used were as follows: solvent A—purified water, solvent B—100 mM NaOH, solvent C—1.0 M sodium acetate in 100 mM NaOH; the solvents were prepared using purified water, degassed by helium sparging immediately before use.

The working temperature of the column was 35 °C and initial equilibration was carried out with 100% solvent B at a flow rate of 1.0 mL min^−1^. Following injection of 25 μL sample onto the column, the compounds were eluted with a 25-min programme based on methods described by Cairns et al. (1999) as follows: a gradient of 100 to 50% solvent B concurrent with a gradient of solvent C from 0 to 50% was applied from 1 to 19.5 min following injection. The percentage of solvents B and C were adjusted to 30 and 70% respectively at 19.6 min following injection, with a further gradient to 100% solvent C achieved at 21.5 min. The column was returned to initial starting conditions of 100% solvent B via a gradient applied over the next 0.5 min and equilibration of the column with 100% solvent B continued for 3 min. In order to estimate the DP of oligomers identified as laminarins in the macroalgal extracts, a range of linear β(1,3) linked β-D-glucans of known DP [laminari-biose, laminari-triose, laminari-tetraose, laminari-pentaose and laminari-hexaose (DP 2-6)] (Megazyme, Ireland) were analysed with HPAEC-PAD using the same parameters.

### Analysis of laminarin extracts by liquid chromatography–mass spectroscopy

The molecular size distribution of laminarin polymers was analysed by reversed-phase liquid chromatography with electrospray ionisation–ion trap tandem mass spectrometry (LC-ESI/MS^*n*^). Samples were analysed on a Thermo Finnigan LC-MS system (Thermo Electron Corp, USA) comprising a Finnigan LTQ linear ion trap with ESI source and a Waters C_18_ Nova-Pak column (3.9 × 100 mm, particle size 4 μm) maintained at 30 °C. Samples were prepared in purified water at 0.2 mg mL^−1^ and 20 μL was injected onto the column. The mobile phase consisted of water/formic acid 100:0.1 (*v*/*v*; solvent A), and methanol/formic acid 100:0.1 (*v*/*v*; solvent B). The column was equilibrated with 98% solvent A at a flow rate of 1 mL min^−1^, with 10% going to the mass spectrometer, and the percentage of solvent B was increased linearly from 2 to 42% over 20 min. MS parameters were as follows: sheath gas 30 and auxiliary gas 15 (both arbitrary units); spray voltage was 4.8 kV in positive ionisation mode, capillary temperature 320 °C, capillary voltage 45 V and tube lens voltage 110 V. The selected mass range was *m/z* 300–4000.

### Enzymatic digestion of laminarin extracts

To confirm that peaks detected by HPAEC-PAD in laminarin fractions were laminarin polymers, crude laminarin samples were digested with laminarinase (Sigma-Aldrich, UK). Laminarinase (1 U mL^−1^) and 20 mg mL^−1^ solutions of laminarins were prepared in 0.01 M/0.02 M McIlvaine buffer, pH 5.0. Each laminarin solution (500 μL) was incubated with 80 μL of 1 U mL^−1^ laminarinase at 37 °C for 240 min. Samples were then heated to 85 °C for 10 min to denature the enzyme. Pure water (420 μL) was added to each sample to give a volume of 1 mL, making the final solution an equivalent concentration of 10 mg mL^−1^ laminarin. Finally, the samples were centrifuged at 8500×*g* for 10 min prior to HPAEC-PAD analysis on a CarboPac PA-100 column as described above.

## Results

**Fig. 1:**
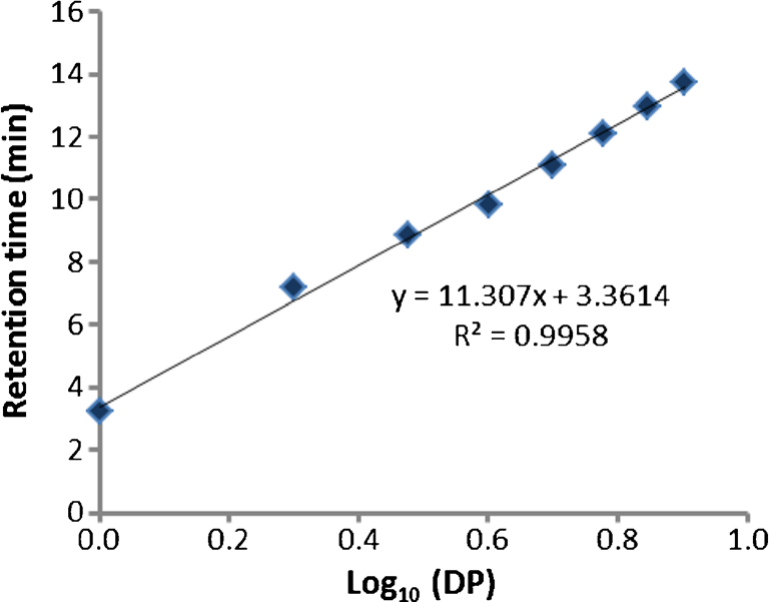
Relationship between retention time and Log_10_ degree of polymerisation (DP) following separation by HPAEC on a CarboPac PA-100 column. HPAEC was carried out with linear β(1,3) linked β-d-glucans of known DP

Laminarin oligosaccharide standards, laminari-biose, laminari-triose, laminari-tetraose, laminari-pentaose and laminari-hexaose (DP 2–6) along with DP 7 and 8 oligomers (which were observed in small quantities in the laminarihexaose sample) were separated by HPAEC-PAD. The retention time for each oligomer is presented in Table 1. When log_10_ values of DP were plotted against retention time, a linear relationship was observed as shown in Fig. 1, represented by the equation

1 $$ y=11.30x+3.361 $$

**Table 1:** Retention times of linear β(1,3) linked β-d-glucans of known degree of polymerisation (DP) following separation achieved by HPAEC on a CarboPac PA-100 column

DP of glucan	Log_10_ DP	Retention time (min)
1	0.00	3.20
2	0.30	7.17
3	0.48	8.84
4	0.60	9.84
5	0.70	11.10
6	0.78	12.13
7	0.85	12.98
8	0.90	13.71

**Fig. 2:**
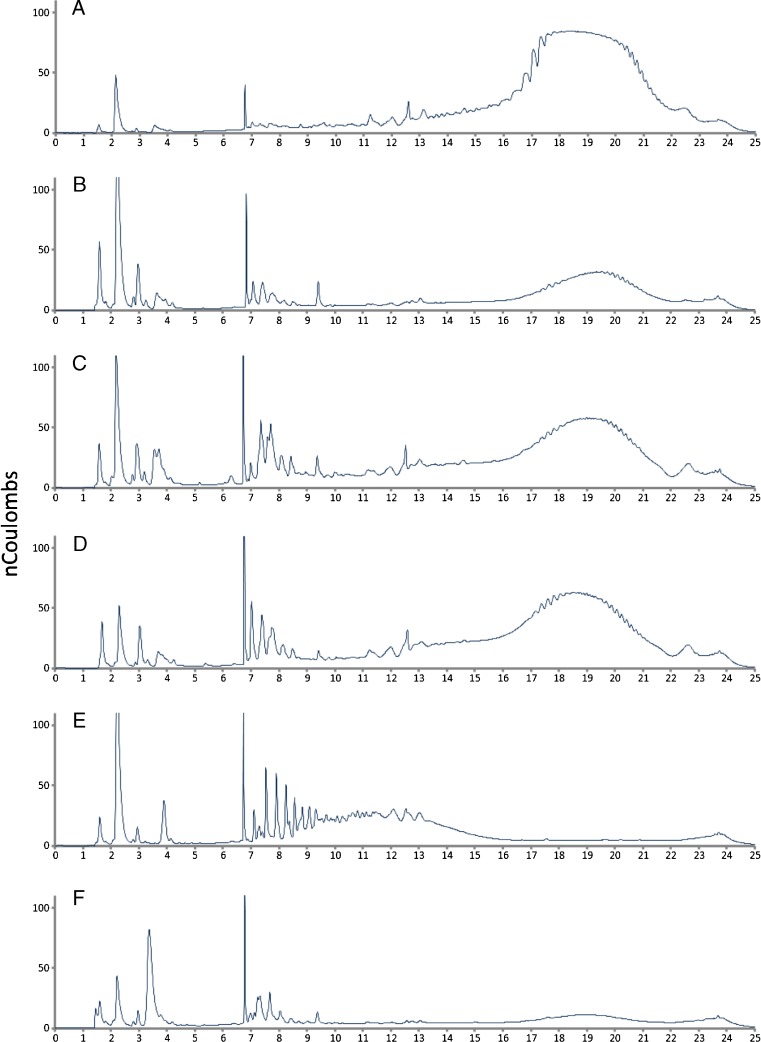
HPAEC-PAD chromatograms showing laminaran extract profiles following separation on a PA100 column: **a** *L. hyperborea*, **b** *F. spiralis*, **c** *F. vesiculosus*, **d** *F. serratus*, **e** *A. nodosum*, **f** *P. canaliculata.* Laminaran polymeric peaks elute between 7 and 22 min retention time. Retention time of coeluting mannitol and fucose is 2.2 min and that of glucose is 3.2 min

Macroalgal extracts were also separated by HPAEC-PAD and laminarin profiles are shown in Fig. 2a–f. Complete removal of these polymeric peaks following digestion by the laminarin-specific enzyme laminaranase confirmed the identity of the peaks detected within the range of retention times 7–22 min as laminarins. Profiles showed variation in size distribution, and the DP size range of the different macroalgal laminarin fractions was estimated using Eq. (1). The laminarin profile for the *L. hyperborea* extract is shown in Fig. 2a with oligomers mainly eluting between 14 and 22 min and the most abundant between 18 and 20 min. Substituting these values into the linear equation gave an estimate of DP ranges of 9–44 and 20–30 respectively. HPAEC-PAD profiles for *Fucus* species, *F. spiralis, F. vesiculosus* and *F. serratus* (Fig. 2b–d), showed a similar pattern indicating a very similar composition and size distribution. The most intense peaks were detected between retention times of 16 and 22 min, with the most abundant eluting at between 19 and 20 min. This corresponds to a DP range of 13–45 and a mean DP size of 24–28. A similar size range was observed with *P. canaliculata* extracts; however, oligomers showed lower abundance. Oligosaccharide peaks in *A. nodosum* extracts (Fig. 1e) showed shorter retention times compared with extracts from the other species. The most abundant polymers were detected between 7 and 15 min which equates with DP2–11.

The mass of laminarin polymers was estimated by multiplying the DP value by 162 Da, which is the nominal mass of a glucose residue. Based on these estimates, a molar mass range of 2000–7000 was predicted for *L. hyperborean*, *P. canaliculata*, and the *Fucus* species. The most abundant *L. hyperborean* oligomers fall in the range of 3200–4800 Da while the most abundant response for *Fucus* species equates to a molar mass of 3900 Da. Calculated masses for *A. nodosum* were lower than for the other brown seaweeds with a range of 340–1700 Da.

Some of the resolved peaks in the lower laminarin DP range showed retention times which matched the laminarin standards; however, several additional peaks were also detected in this range. Peaks which elute earlier than laminarin oligomers are also evident in the chromatograms. The monosaccharides mannitol and fucose were detected in laminarin extracts analysed by HPAEC-PAD. Injection of standard solutions of monomeric sugars demonstrated that the mannitol and fucose coeluted under the chromatographic conditions used in HPAEC-PAD analyses, with a retention time of 2.2 min. Glucose eluted with a retention time of 3.2 min. Laminarin samples contained low concentrations of glucose as shown in Fig. 2. Mannitol/fucose peaks with signal strength over 150 nC were detected in *F. spiralis* and *A. nodosum*; a peak with signal strength of approximately 110 nC was detected in *F. vesiculosus* and peaks of <50 nC were detected in *P. canaliculata*, *F. serratus* and *L. hyperborea* (Fig. 2).

**Fig. 3:**
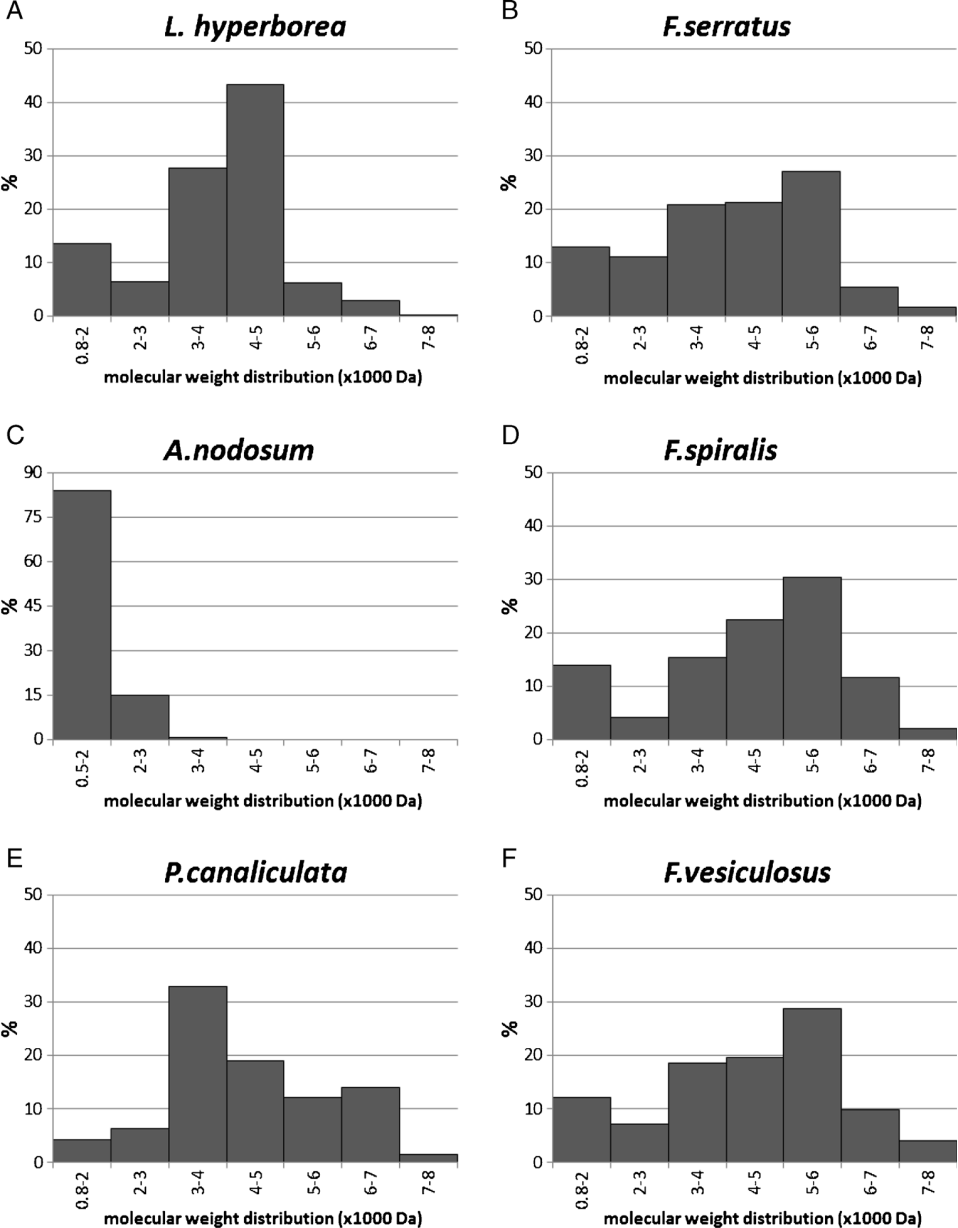
Molecular weight distribution of laminarins in seaweed extracts determined by LC-ESI/MS^*n*^: **a** *L. hyperborea*, **b** *F. serratus*, **c** *A. nodosum*, **d** *F. spiralis*, **e** *P. canaliculata*, **f** *F. vesiculosus*

Laminarin extracts were analysed by LC-ESI/MS^*n*^ to determine the molecular weight distribution by an alternative method. Measurement in full positive MS mode produced spectra showing three distinct sets of ions which increased by increments of 162, 81 or 54 *m/z* units corresponding to single-, double- and triple-charged glucose polymer series respectively (Online Resource 1). Peaks were generated by restricted selection of laminarin ion *m/z* values, and these were integrated to estimate abundance up to a molecular mass of 12,000 (triple-charged ions at *m/z* 4000) (Fig. 3). The data shows that the majority of the laminarin fraction in *L. hyperborea* and *Fucus* species extracts fall in the range of 3000–6000 Da. The most abundant ions in these extracts corresponded to a DP of 25 for *L. hyperborea* and DP 29/30 for *Fucus* species. *P. canaliculata* showed a more even distribution over the 3000 to 7000 Da size range. A distinct profile was observed for *A. nodosum* extracts which showed a much lower size distribution with the bulk of the fraction detected below 2000 Da and the most abundant ion corresponding to a DP of 6.

## Discussion

Analysis of laminarin fractions extracted from a range of brown seaweeds with the Dionex method described here yielded results which compared well the molecular size distribution determined by the LC-ESI/MS^*n*^ method. The observation of multiple charged species in MS spectra is consistent with the findings of Graiff et al. (2016) and enabled analysis of larger species by the ESI/MS method. The data reported here is also in good agreement with values reported in the literature. Kadam et al. (2015) analysed *L. hyperborea* and *A. nodosum* laminarin extracts using MALDI-Q-TOF-MS to determine the molecular weight distribution. Their study showed that *L. hyperborea* laminarins ranged from 3243 to 5052 with a DP range of 20–31, which is in close agreement with values obtained in the current study for the most abundant fraction in extracts from this species (DP 20–30; molar mass range 3200–4800 Da). In a similar study, Chizhov et al. (1998) determined a mean DP value of 26 for laminarin from *L. hyperborea* with a maximum DP of at least 40 using MALDI and FAB MS. Characterisation of laminarins by Graiff et al. (2016) confirmed a molar mass distribution of 2000 to 7000 Da for a commercial laminarin from *L. digitata*, and this correlates with the size range determined for brown seaweed extracts determined in the current study*.* These authors also reported molar masses ranging from 2400 to 4400 Da for *Fucus* species which is broadly in the same range as the values determined in this study where the most abundant fraction is predicted to have a molecular weight of 3900–4590 Da. Results for *A. nodosum* laminarin obtained by Kadam et al. (2015) are in contrast with values determined by the calibration method described above. These authors report DP values in the range of 25–30, which greatly exceeds the size range of 2–11 determined in the current study. It should be noted that the structure of laminarin is influenced by environmental factors, such as water temperature, nutritive salt, salinity, waves and sea current (Rioux et al. 2009), so some variation may be attributed to differences between extracts used in this study which may account for the observed differences. Efficiency of extraction may also be a factor; however, the extraction process described here is based on solubility in aqueous medium, and Graiff et al. (2016) concluded that laminarin from *L. hyperborea* and three *Fucus* species was completely water-soluble and fully extracted in cold water. It must be noted that analysis of size distribution by the Dionex method is based on an equation generated with standards restricted to a maximum DP of 8. There is a high risk of error extrapolating to much higher DP values and ideally, retention times of oligomers of higher DP should be included to generate a more accurate equation. Notwithstanding these limitations, the data showed good agreement with values estimated by LC-ESI/MS^*n*^. Moreover, the similarity in DP and peak abundance between this and other studies for the other laminarin fractions adds further support to the robustness of the method reported here.

The observation of extra resolved peaks within the retentions times associated with low DP oligomers suggests that these early eluting oligosaccharides are being selectively separated due to other physical characteristics than purely degree of polymerisation. Branching and linkage arrangement influences the retention time of carbohydrates on CarboPac PA100 columns with disaccharides (cellobiose (β-1,4 linked beta glucose monomers), maltose (α-1,4 linked beta glucose monomers) and laminaribiose (β-1,3 linked beta glucose monomers) eluting with varying retention times (6.07, 6.77 and 6.92 min respectively). This suggests that the peaks detected between 7 and 9.5 min indicated low DP laminarins but with a degree of branching or the presence/absence of mannitol terminal residues.

Differences in WSC composition reflected the taxonomic separation at the order level for the macroalgae chosen for this study. It is known that there is a diverse range of polysaccharides in macroalgae which vary both between and within algal taxa (Stengel et al. 2011). All the selected samples are from the class Phaeophyceae. *L. hyperborea* is from the order Laminariales, whereas all other algae are members of the order Fucales, family Fucaceae. Within this family, *P. canaliculata* and *A. nodosum* are separated from *F. spiralis*, *F. vesiculosus* and *F. serratus* at the genus level.

In conclusion, application of the HPAEC-PAD method as described demonstrates a semiqualitative method for characterisation of the structural variation and DP of laminarins extracted from brown macroalgal species harvested from the Ceredigion coast. Estimates generated by this procedure showed good agreement with analysis by LC-ESI/MS^*n*^ and were generally consistent with data reported in the literature. The method does not provide information on the presence of mannitol or branching frequency which can be obtained using derivatisation coupled with acid hydrolysis and gas chromatography-MALDI-TOF analysis. However, as an initial analysis, it provides a simple, convenient, rapid and semiqualitative indicator for the range of DP of laminarins present in the macroalgal samples, informing their suitability as potential nutraceutical compounds.
